# Factors that influence data sharing through data sharing platforms: A qualitative study on the views and experiences of cohort holders and platform developers

**DOI:** 10.1371/journal.pone.0254202

**Published:** 2021-07-02

**Authors:** Thijs Devriendt, Pascal Borry, Mahsa Shabani

**Affiliations:** 1 Faculty of Medicine, Department of Public Health and Primary Care, Centre for Biomedical Ethics and Law, KU Leuven, Leuven, Belgium; 2 Faculty of Law and Criminology, Metamedica, Ghent University, Ghent, Belgium; University of Rennes 1, FRANCE

## Abstract

**Background:**

Infrastructures are being developed to enhance and facilitate the sharing of cohort data internationally. However, empirical studies show that many barriers impede sharing data broadly.

**Purpose:**

Therefore, our aim is to describe the barriers and concerns for the sharing of cohort data, and the implications for data sharing platforms.

**Methods:**

Seventeen participants involved in developing data sharing platforms or tied to cohorts that are to be submitted to platforms were recruited for semi-structured interviews to share views and experiences regarding data sharing.

**Results:**

Credit and recognition, the potential misuse of data, loss of control, lack of resources, socio-cultural factors and ethical and legal barriers are elements that influence decisions on data sharing. Core values underlying these reasons are equality, reciprocity, trust, transparency, gratification and beneficence.

**Conclusions:**

Data generators might use data sharing platforms primarily for collaborative modes of working and network building. Data generators might be unwilling to contribute and share for non-collaborative work, or if no financial resources are provided for sharing data.

## Introduction

Data sharing in science maximizes the utility and impact of patient data, and therefore contributes to improving clinical practice and public health. In practice, this means that data can be used to explore ideas not envisioned by the data generators, to perform co-analyses with higher statistical power, to replicate or validate findings through different statistical methods or to educate students [1–4]. Nevertheless, the academic literature has recorded a range of reasons why researchers do not share data. These include the fear of getting “scooped” and not being sufficiently attributed, the loss of intellectual property rights, misinterpretation or misuse of data, the lack of resources or expertise to prepare data for sharing, confidentiality of data and privacy concerns, the lack of (free-of-charge) infrastructure for sharing, fear that re-analysis of data might invalidate earlier results, institutional policies, regulatory limitations and the absence of uniform research policies [5–8]. While researchers consider that the sharing of data can have some positive effects on career advancement, such as establishing collaborations and increasing visibility for the research team, the lack of incentives for sharing has been emphasized by academics and policy makers and is often conceptualized as the lack of status/recognition conferred on those who share data [6,8–12].

Aside from the barriers documented for data sharing in general, there is some literature that describes the struggles of data sharing infrastructures to incentivize data contribution by those that generate data (hereafter “data generators”). For example, the WorldWide Anti-malerial Resistance Network (WWARN) data platform reported that data generators only agreed to contribute data after being promised co-authorship on papers in high impact factor journals for data usage [13]. At a later stage, publications of pooled analyses performed by WWARN followed the ICMJE guidelines more strictly, although this move was met with dissatisfaction on part of some data generators [14]. Murtagh *et al*. describe four areas on which progress is needed in order to establish functional biomedical data platforms. Two out of those areas emphasize elements related to credit and recognition of scientists such as *“[the] recognition of the investment of scientists [for generating data] (…) [and] the substantive contributions of everybody in building*, *maintaining and operating data infrastructures”* [15].

In 2016, the European Commission has launched several initiatives to establish novel data sharing platforms in the areas of epidemiology, such as euCanSHare, CINECA and EUCAN-Connect, which aim to speed up cohort browsing and data access procedures, management and analysis. In light of previously reported difficulties to motivate cohort holders to share data through platforms, the aim of this study is to describe the factors that influence decisions on data sharing in greater contextual depth. The general objectives of this interview study were to (1) document the views and opinions on different incentives for data sharing; (2) explore past experiences on data sharing and crediting mechanisms within consortia; (3) record views on the roles of different actors within academia to change the existing incentive structure for data sharing; and (4) investigate the interaction between data sharing practices and novel technologies. This article outlines factors that influence data sharing based on the collected data pertaining primarily to previous experiences with sharing. Results based on another subtheme of the categorized data, incentive mechanisms for sharing (in terms of credit), will be reported and discussed elsewhere due to the different thematic focus and the excessive wordcount reached when integrating the two texts.

## Materials and methods

Qualitative methods were used to explore the views and opinions of cohort holders and platform developers. The approach taken towards data collection was the case study, which allows to holistically study data sharing experiences in greater contextual depth. Cohort holders were defined as those that manage access to cohort data and have often been involved in data generation. Platforms developers were considered to be those involved in designing components (e.g. catalogue, analytical toolbox) of data sharing platforms. Most participants could be classified as cohort holder, or both cohort holder and platform developer. Cohort holders were primarily recruited because of their previous experiences with data sharing and their views on submitting data to platforms. Platform developers were recruited due to their experiences with difficulties to keep cohorts engaged, and potential avenues to address these problems through technical means. They were recruited using a purposive sampling strategy exploring three European projects creating data sharing platforms (euCanSHare, CINECA and EUCAN-Connect) [16–18]. These three projects are all funded under the same H2020 call. Contact persons for cohorts within the euCanSHare consortium were identified via a list of names acquired from the project manager and other lists found online. Within euCanSHare, nearly all European cohorts were contacted, except those where the PI had passed away or where multiple cohorts were managed in the same center. Names of contact persons from the CINECA and EUCAN-Connect consortium were acquired by either contacting the project coordinators or by querying participating cohorts in databases and registering the first or last authors on Cohort Profiles or recent articles. Potential interviewees were contacted by email. Interviews were semi-structured in nature and were conducted by TD, using an interview guide. TD has a background in biomedical sciences, although not epidemiology specifically. TD has no personal relationship with any of the participants. Several participants pointed out that TD was responsive and guiding conversations well, with one participant raising that TD interrupts rather quickly. In general, TD holds the assumption that researcher behavior is partly steered by what is rewarded or what is commonly seen as valuable, institutionalized through social structures in science. The interview guide was slightly altered after conducting the first interview (e.g. redundant questions were removed). All interviews were performed between December 2019 and June 2020. Data collection was temporarily suspended for two months due to the COVID pandemic. In total, seventeen interviews have been conducted; thirteen with cohorts affiliated with euCanSHare and four with other data sharing platforms. Sampling saturation was found after approximately thirteen interviews within the euCanSHare consortium. Further interviews with non-euCanSHare participants indicated the existence of different “subcultures” within cohort research. Interviews were audio-recorded, transcribed verbatim, de-identified and analyzed using inductive content analysis in which content categories are derived from the data, rather than pre-determined [19–21]. Transcripts were coded into narrow content categories using NVivo 12 software by QSR International. Subsequently, categories were compared, revised and broadened through iterative exploration of excerpts, the coding scheme and original transcripts. Upon completion, the coding scheme and extracts were checked by MS for consistency and rationale. The coding scheme went through several iterations after being discussed with MS and PB. During the development of the coding scheme, the terms “incentives” and “disincentives/barriers” were collapsed into the more neutral term “factors” due to (a) disincentives/barriers not capturing socio-cultural factors and objection being raised to classifying ethical elements as such; and (b) categories for disincentives and incentives being coded in duplicate as they are opposites (e.g. quotes on lack of credit and acquiring credit overlap and were covered in separate categories). All participants signed a written informed consent. Interviews were conducted online (Skype for Business, Zoom, GoToMeeting, Google Meetings) or via phone and recorded. Skype for Business was preferred due to institutional support, although many technical difficulties occurred which necessitated the use of other programs. The study was approved by the Social and Societal Ethics Committee (SMEC) at KU Leuven (G-2018 10 1348).

## Results

**Fig 1:**
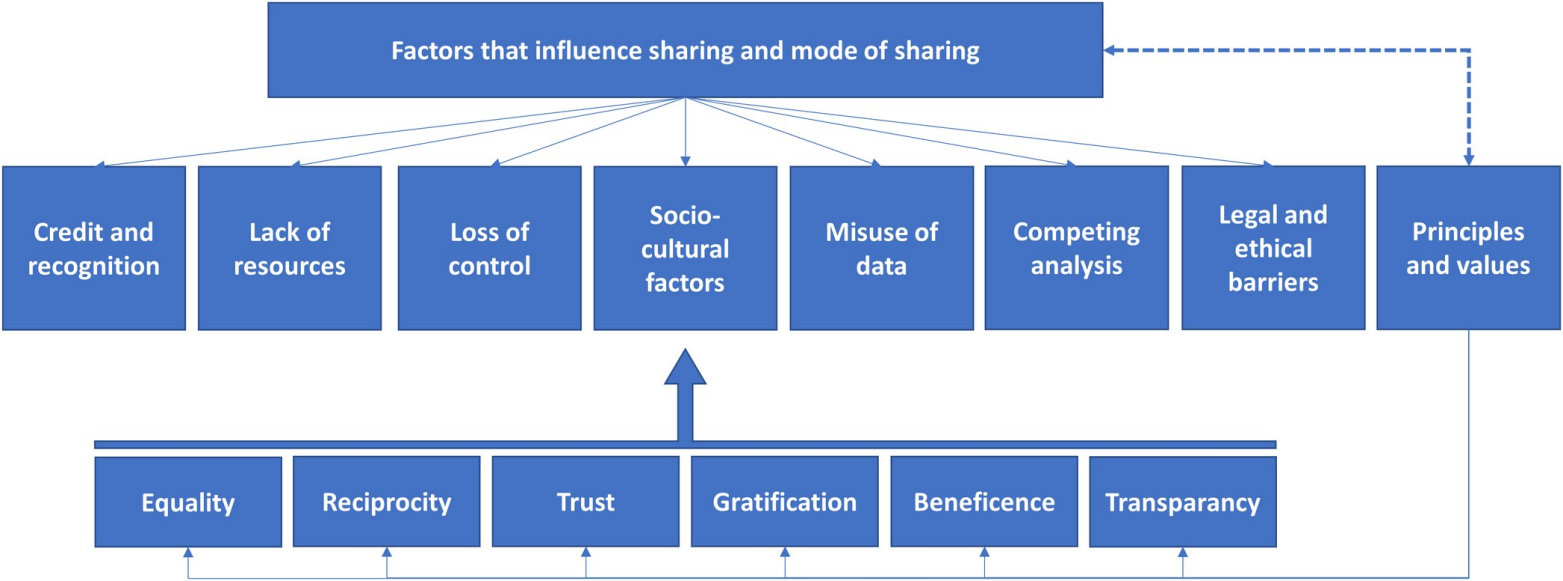
Coding scheme derived from interview data.

Data collected during the interviews were classified and the following two categories emerged: (a) factors that influence data sharing and mode of sharing and (b) core values and principles to data sharing. A tree diagram of the main categories and all subcategories is displayed in Fig 1.

### a. Factors influencing data sharing and mode of sharing

**Fig 2:**
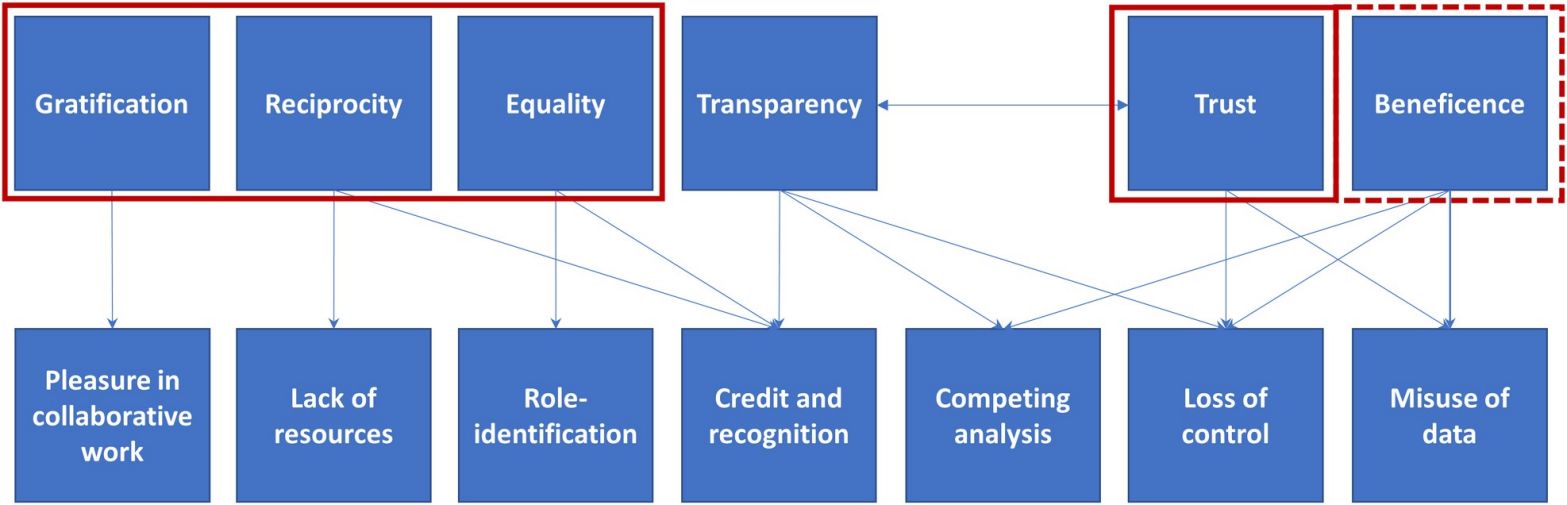
Core values and principles underlying factors influencing data sharing. Values that underpin data sharing were identified from arguments in the interview data. No connections were coded where arguments were absent but where connections could theoretically exist. Competing analysis was coded as a separate category due to its connections with various values. Socio-cultural factors were split into role-identification and enjoyment in collaborative work. Values which push researchers towards collaboration are framed with red boxes.

The interviews showed that a broad range of factors influence scientists’ decisions regarding data sharing. These factors can be categorized as follows: (1) credit and recognition; (2) potential misuse or misinterpretation of data; (3) lack of resources; (4) loss of control; (5) socio-cultural factors and (6) ethical and legal barriers. Throughout the interviews, respondents put forward several values and principles that they considered essential when sharing data and which influence data sharing behavior. They were categorized as follows: equality, reciprocity, transparency, trust, beneficence and gratification. These values could be linked to the aforementioned factors (Fig 2).

#### 1. Credit and recognition

The first factor is the extent to which the original team is involved in downstream analysis. Data may be shared for collaborative analysis, with varying degrees of involvement of data generators, or without active involvement at all. The vast majority of interviewees consider active collaboration to be the most desirable mode of working when sharing cohort data and several participants would require active collaboration as a precondition for sharing.

*“[We look at if] there is a sufficient involvement of expertise from our own institution. (…) While we share data, we do not just give it away. This means [if] someone from Greece or from Spain (…) looks in our system and applies for data, that person will not get anything. It depends on some feedback with our internal collaboration with the [appropriate] background.” (Interviewee 6)*

Active collaboration is preferred by many interviewees in order to safeguard the academic interests of data generators, and to ensure correct scientific interpretation of the data. Assembling cohort data can take more than ten years, from the conception of study design to data generation, and finally to quality assessment. During this period, interviewees argued that scientists dedicate their time and resources to these processes, and pay the opportunity cost by missing out on potential publications. Later, updating the cohort with follow-up data/information such as deaths, disease events or novel examinations was considered to generate a long-term workload for data generators. Therefore, some interviewees argued that the creation of datasets serves as an investment to later publish academic papers and establish collaborator networks. Without this pay-off, it is argued by several interviewees that data generation would not be interesting, as one could simply use others’ data. Additionally, the preparation of datasets before sharing might still take considerable time if some data need to be extracted from the public health system or *ad hoc* anonymized datasets need to be generated for project proposals. In view of this workload, multiple interviewees raised the concern that data contribution without active involvement is presently not adequately recognized.

*“It is also safeguarding rights and interest because after all, if [cohort] data gets published without anyone involved from us, we are not counted and just providing data has zero value.” (Interviewee 6)*

An exception may exist for the sharing of data if it does not result in direct strategic conflict. For example, research teams might share genetic data for genetic epidemiology studies when they do not have expertise in that area.

In case data requests are received for competing analyses, most respondents answered that they would not provide data. A range of solutions would be proposed to resolve this situation including (a) collaboration between the two groups; (b) steering the most recent proposal towards a different focus point; or (c) conducting an independent analysis using different methods. Respondents generally held the view that the replication of studies in this way goes unrewarded in the academic system.

#### 2. Misuse or misinterpretation of data

Furthermore, the majority of interviewees consider that it is advantageous to involve data generators as they are knowledgeable about the study design and its limitations, how variables were generated, and the laboratory and quality control procedures. One interviewee also argued that there is the possibility of encountering unexpected findings in the results. This would necessitate dialogue with the data generators to explore whether certain particularities in the data generation process could explain these findings. The sharing of data without active involvement is considered by some to enable interpretations that may not be scientifically valid. Misinterpretations may occur on the level of study design, the meaning of variables or how to appropriately address biases in the dataset. Complex or context-specific variables, older or research-driven cohorts with unique study designs are argued to be more susceptible to misinterpretation. Several interviewees could name specific instances where misinterpretations have occurred.

*“We have a group working on one of our datasets and they have used some of our variables in a wrong way. We are in a discussion with them because we want them to redo the analysis and they do not want to. (…) We do not want them to publish because they have used the variables wrongly. (…) It is [an] educational variable. (…) The [local] education system is not like [theirs] so they have misinterpreted the way they are generated and what they mean.” (Interviewee 11)*

Data dictionaries, which include metadata on variables, may to some extent facilitate reuse without active involvement. Nevertheless, some participants considered that studying this document was time-intensive and may not resolve all questions data analysts have.

#### 3. Lack of resources

Interviewees argued that scarcity of resources is a barrier for data sharing. Sharing requires pro-actively thinking about organizational elements, such as ethical and legal provisions, setting up Data Access Committees, and establishing access systems for data requests. Additionally, one should collect sufficient documentation on study design choices, variable construction and their underlying rationale, which of the original research questions could and could not be answered, and limitations related to data quality. One interviewee argued that these elements related to long-term sustainability of the cohort are not prioritized when resources are scarce. Moreover, the act of making cohort data itself available is argued to take a considerable amount of time.

“*Just looking at the access application and working out*: *do we have the data*, *how can we support [the proposed study]*, *how much would it realistically cost to make this available*, *is actually a massive opportunity cost*.*” (Interviewee 12)*

The time spend on this step might be substantially reduced when datasets are already harmonized within consortia, and when the consortium has a centralized committee to examine the scientific validity of the proposals.

Some interviewees argued that when funding is acquired, for example through consortia, the person hired for that project can make the data available for sharing. When no financial support is available however, research teams have to drain their own resources, while not being adequately rewarded. One interviewee thus argued that to keep access to the data resource affordable in the long-term means that groups might ask for standardized or *ad hoc* fees to hire personnel for the preparation of anonymized datasets.

#### 4. Loss of control

Another important condition that influences data sharing behavior is the desire to remain in control of downstream data usage and to enforce publication or funder policies (e.g. acknowledging funders). Some interviewees expressed concerns that by sharing data, they may lose control on the downstream data uses such as the use of data for competing analyses without their knowledge. Some interviewees feared loss of control during the pre-publication process: the loss of opportunity to verify whether the research is in line with the consent, to check whether the dataset is appropriately anonymized or whether particularly sensitive data is disclosed (e.g. certain survey data).

*“Once we release it, [we need to] maintain control over it. (…) A problem with data sharing is study overlap, it is keeping track of who is doing what with the data. (…) We are hoping that [if] anybody accesses data through these platforms, we will still be informed of what research is taking place. (…) There are certain elements in the X study where we were capturing sensitive issues [related to regional conflict] in the data. We cannot release that due to the sensitivity and because of the potential disclosure of individuals from that. (…)” (Interviewee 9)*

Furthermore, there are concerns over the ability to enforce certain policies (e.g. formal publication policies of cohorts or funder policies) during the publication process. For example, interviewees wanted to see any outputs and check that (a) necessary authors were included; (b) no persons are potentially re-identifiable; (c) research participants are properly acknowledged and (d) a funders statement is included.

#### 5. Ethical and legal barriers

Complying with the requirements set by the General Data Protection Regulation (GDPR) and ethics committees (e.g. in terms of informed consent) was considered to bring several challenges to data sharing. The principle concerns included the increased bureaucracy brought to academics, divergent interpretations of GDPR between institutions and countries, an overly strict standard of re-identifiability that is used and the validity of older informed consent forms. Interviewees communicated that compliance with the relevant legal provisions requires administrative support in order to keep the workload manageable. Some interviewees stated that they are not sufficiently assisted by the legal services of the institution, in which case the bureaucratic load falls entirely upon themselves. Several respondents considered the retrospective application of requirements for informed consent under GDPR to be problematic. As older cohort studies might have collected consent forms in manners that do not comply with the current regulations, their usage for research might be challenged.

*“We have had collaboration with another research group in Australia. Recently, the data access agreement expired (…) and we tried to renew it. The legal team of [our institute] said that it needs to be the controller-to-controller version of the EU contract clauses and the legal team of the other institute said that they could not sign such an agreement. There were disagreements basically on the [precise wording] of the agreement, but [our institute] cannot easily change the wording of these clauses. We had to cut the data access for the Australian team although we have worked together without problems for ten years. (Interviewee 2)*

#### 6. Socio-cultural factors

Notably, several interviewees explained their unwillingness to share as data contributors by referring to their identity or role as scientists, or to their enjoyment in collaborative modes of working. Only sharing data is not considered to be intellectually stimulating by researchers. Moreover, co-authorship on papers in prestigious journals is then not considered of much personal value. The lack of opportunity to contribute to the development of the data analysis plan when sharing data is described by some as somewhat unfitting for scientists. In contrast, several participants vividly described their personal satisfaction with actively exchanging ideas and methodologies, getting to know other scientists, and the feeling of *“being in this together”*.

*“We are scientists so we want to participate in research so (…) just sharing data can be a matter for people that produce data, without any research activity. Perhaps for many clinicians that just provide data on their patients, but they have no interest in the research. (…) I am not so happy to have ten names in Nature, without participating in any way to the idea, the analysis or the discussion. I am a scientist so my place is to produce research by myself” (Interviewee 13)*

### b. Core values and principles in data sharing

Equality refers to partners feeling that they are being treated as equals, as peers. Equality influences the self-perceived identity of researchers as one may feel equal only when fulfilling the “proper” role of a researcher. If leading analyses is seen as the pre-eminent component of that role, the identity is threatened when researchers engage in unilateral sharing. Furthermore, lack of recognition also can make one feel as not being an academic on the same level.

“*We do not like to seek collaborations in which we cannot be an active partner*. *We want to create a real network*, *a real collaborative group*. *Our policy is not to be seated upon hundreds of tables without the opportunity to speak or to give our opinion*. *I mean to feel as peers in collaborative research*. *It is something that we have always found in consortia X and Y” (Interviewee 7)*

Reciprocity refers to the fact that no party is unjustly disadvantaged in terms of academic credit by contributing data: When one shares, all others must share as well. If data is shared without collaboration, one is disadvantaged as one is, in comparison to the others, insufficiently rewarded. Unrestricted sharing of data could undermine the rationale for generating data in the first place: If all others share, then researchers could simply reuse others’ data. Lack of resources relates to reciprocity because when one invests resources in data sharing, it comes at the expense of other activities. If data sharing is then less rewarded in comparison with these other activities, researchers may put themselves at a disadvantage.

*“If we were just giving our data away openly… we [emphasis] open up the study to others, period. It would be likely that within a few years, it might become a threat to the existence of the study itself. (…) It is the incentive system: Are the researchers able to acquire what they need from the projects they set up? (…) You might have other researchers that are just waiting and when the study is done, you open up the data and they can just jump on it and work with it. If two years after that, all those groups, they have done nothing else than publishing, they will be the ones that are seen and there is no way to credit those that have created the data.”*

Transparency refers to the need for clear rules on the ability to participate in downstream analysis, on providing input at various stages and on publication and authorship policies. Scientists need to be able to fully observe the conditions under which data are shared, and obscurity is considered detrimental to scientific collaboration. Lack of transparency contributes to the feeling of losing control; one cannot predict what will happen and what consequences it will have for the team.

*“We had a very smooth collaboration and continued to collaborate after the end of the project. The main reason was that the process was transparent, and all the participants were put on the same level. (…) We had very clear rules for data sharing, for publications, for names on the papers and we feel good with this process.” (Interviewee 13)*

Within this context, trust can be conceptualized as the belief in the reliability of others to adhere to established agreements, and to behave rightly in those situations where agreements have not been established. Data contributors need to be able to trust in the competency of all members of the research team contributing to the analysis and in them treating the data with proper care. Lack of trust then contributes to loss of control; before data can be shared, trust must be established between parties.

*“Something which is very important is trust. That those who are sharing the data are always sharing it for collaborative research [and] that they can trust that the data is treated as it should be treated. (…) When we send data to a new place or in particular to some collaborator who is not a member of our consortium, that they hesitate to allow to use or transfer the data. (…) There seems to be very strong resistance from the [cohorts] because they do not yet trust that place.” (Interviewee 1)*

Beneficence (or “goodness”) is to be understood as what is good for science, for patients or for society. Beneficence is strongly linked to misuse of data, as such use would result in erroneous research results. This is argued by some to be harmful down-the-line for trust in science and public health policy. Loss of control includes losing the ability to carry out re-identifiability checks and protect patients’ interests, and may therefore be harmful. Finally, beneficence heavily influences the perspectives on competing analyses; some held that (forms of) competing analyses would benefit science, while others claimed that does not benefit science as it creates redundancy too early in the scientific process.

*“These studies are really complicated, they often have complex study designs and different drop-out dynamics. Some [research] questions that were asked did not work, others worked better. Some were understood, others not understood and it is very difficult to just hand that over to someone else without all the background information. (…) I do not mind people using my data but [they should] understand how the data was generated and what the limits were. “Take this [dataset] and do what you [want]”. I am not against that principle. I hope that we will get there but we are not there yet.” (Interviewee 17)*

Gratification is the value that pertains to experiencing enjoyment in the scientific work. Collaborating when sharing data is seen as a social process in which one finds enjoyment.

*“In consortium X, we start from a proposal where we describe the aims and what the main analysis will be, the expected results and so on. Then we try to send technical details to statisticians so we can have feedback on the methodology and then we have several rounds of drafting. (…) Collaborating in [an active] way is much more stimulating than just to be data provider.” (Interviewee 7)*

## Discussion

Interviewees outlined their past experiences with data sharing and described the elements which they perceived as impediments (see S1 Table). In this way, a range of factors that influence individuals’ decisions to share data, and their preference regarding the mode of sharing were identified, such as academic credit and recognition, lack of resources, misuse or misinterpretation of data, loss of control, socio-cultural aspects and ethical and legal barriers. Based on arguments provided by interviewees, the values underlying these factors were classified as equality, reciprocity, transparency, trust, gratification and beneficence. The majority of factors can be argued to push researchers towards collaborative modes of working. By the same token, most core values and principles steer researchers towards collaboration, with the only exceptions being transparency and beneficence. While transparency is strictly speaking neutral towards the mode of working, arguments related to beneficence are invoked both in favor of and against broad data sharing. No noticeable differences were observed between interviewees at different career stages (junior vs. senior).

Academic credit and recognition, loss of control, lack of resources, misuse of data and ethical and legal barriers are all factors that have been documented within the literature [5–8]. In order to properly understand the concerns around academic recognition, two premises must be made clear. First, the production of data is an investment for the publication of articles. Thus, sharing without being personally credited creates a free-rider problem, where more scientists are using the resource than the number that are contributing to its production in terms of the investment of time and resources. The sensitivity of not collaborating with data generators is reflected in one editorial by Longo *et al*. in the *New England Journal of Medicine*, which referred to scientists who engage these practices as “research parasites” [22]. This characterization was criticized within the bio-informatics community as restrictions to broadly share data are argued to hamper scientific progress [23]. Second, the act of sharing data meaningfully requires time and resources itself. The importance of this second aspect is diminished if the cohort possesses an elaborate bureaucracy to handle data sharing efficiently. Simell *et al*. have therefore pointed out that the model of cohort access governance where local scientists are in charge of both administrative aspects of sharing and of conducting analyses has rate-limiting effects on data sharing [24].

Other factors put forward by interviewees, such as the fear of misinterpretation, reinforce the preference for collaborative approaches over sharing data without active involvement. Researchers might worry that the study team would incur reputational damage when data is being used in an improper manner [6,8,9,25,26]. The concern over wrongful interpretations also relates to the complexity of observational research. Harper described how the various methodological choices, such as regression specifications, coding and inclusion of covariates and decision rules, can lead research teams to find conflicting results when starting from the same hypothesis and dataset [27]. Rolland *et al*. documented the decision-making processes within collaborative cancer epidemiology research when post-docs were provided with “Small Data” datasets [28]. Their work indicates that analysis pipelines are highly iterative and explorative, with many rounds of consultation necessary with data generators to understand correctly the variable coding procedures and their rationale, the study design or how to correct for potential biases. Therefore, they underline the necessity of collaboration when analyzing small datasets and suggest thoroughly documenting variable coding procedures and decision histories. Nevertheless, some have urged that although this risk of misinterpretation exists, broad sharing of data outweighs these risks by enhancing the production of better research in general [23].

The fear of scientists about losing control of data usage and the lack of resources has previously been documented [5,6,8,29–33]. The importance of broader cultural changes in data sharing practices have been underlined in many articles [9,11,34,35]. Role-identification could be considered such a cultural barrier. Within identity theory, role-identities represents the set of internalized meanings associated with a role [36]. People then make behavioral choices that are aligned with these underlying identities. From this perspective, data sharing behavior might be impeded if scientists consider that data sharing is not part of their role, while leading analysis constitutes their most important activity, especially if the former comes at the cost of the latter. Over time, this understanding of roles cannot be maintained as science advances along the path of specialization towards increasingly collaborative, team-based science. Therefore, actual scientific roles of individuals are bound to become increasingly specialized and diverse.

Values and principles for data sharing have primarily been studied in the context of partnerships in multi-center international health research between high-income countries (HICs) and low to middle-income countries (LMICs). Reciprocity is often phrased as an essential value when sharing data, and ideal relationships between researchers when sharing data should be reciprocal and mutually beneficial [9,22,37]. Lack of reciprocity is then considered to cause an unfair distribution of benefits [38]. The importance of equal partnerships and personal relationships has also been emphasized [38,39]. One document proposes that “equity” (i.e. equality of opportunity) rather than “equality” itself is crucial, with inclusion (e.g. in downstream analyses) falling under the former [37]. Trust and transparency are also widely considered key factors that enable collaborations [37–40]. Within the context of biobanks, Hoeyer *et al*. has proposed that limited sample sharing can be ascribed to the lack of trust rather than concerns over recognition [41]. This indicates that the values and principles that underpin data sharing are not exclusive to one context. Instead, they are likely commonly shared values that instruct the conception of proper behavior when sharing data even within high-income countries.

In conclusion, these results indicate that data sharing platforms might be primarily used by scientists for collaborative modes of working and network building. One example is to utilize the platforms to seek comparable datasets for meta-analysis or validation of hypotheses. This would, in principle, not maximize the potential of the platforms. Designers of data sharing platforms might wish to find solutions for commonly reported problems, such as concerns over credit and recognition, lack of resources or legal barriers.

## Conclusions

Data sharing platforms might have difficulties attracting data generators to contribute data without restrictions, especially where non-collaborative modes of working are envisioned or where scientific work is not financially compensated for the data generators. The widespread usage of platforms to share the most valuable datasets for *all* who wish to utilize them is therefore unlikely to be realized. Concerns over credit and recognition, misinterpretation, loss of control, lack of resources, socio-cultural factors and ethical and legal barriers may influence decision on data sharing *and* the mode of sharing. Reciprocity, equality, transparency, trust, beneficence and gratification were identified as values that underpin data sharing behavior.

## Limitations

Many of the interviewees recruited actively participated in collaborative research within existing consortia. Therefore, positive experiences with such modes of working might have steered the opinions in favor of active collaboration. With cohorts outside of euCanSHare, some interviewees held more heterogeneous views, especially when data generation was funded for the principal purpose of sharing data broadly, when already having experiences with progressive evaluation criteria or when faced with higher degrees of centralization of research governance. As such, the discussion on incentives for data sharing is likely to be *highly* context-dependent, with cultural differences existing even within different branches of cohort research, and researchers being subject to different national, institutional or departmental regulations and rules.

Within the interview study, information related to the role of different entities in changing the incentive structure was variable and not always informative. Due to the variety of barriers and positions identified, synthesizing concrete actions to be undertaken by actors was difficult. The relationship between data sharing practices and novel technologies was difficult to assess as technological innovation was not brought up in various interviews. Additionally, researchers were not always aware of the details of these technologies and their impact (e.g. on data protection regimes and the administrative burden).

## Supporting information

S1 Table Summary table of answers to questions on experiences with data sharing. (DOCX)

S1 File SRQR checklist. (DOCX)

S2 File Interview guide. (DOCX)
