# Supplementary material for: Factors that influence data sharing through data sharing platforms: A qualitative study on the views and experiences of cohort holders and platform developers
Source: PLoS One. 2021 Jul 2;16(7):e0254202. doi: 10.1371/journal.pone.0254202 (PMC8253381; doi:10.1371/journal.pone.0254202)
Supplement: S1 Table — (DOCX) [file pone.0254202.s001.docx]

| *Table 1. Experiences of cohort holders and platform developers* | | | |
| --- | --- | --- | --- |
| Interview # | Shared through international consortia | Shared through biobank | Responses to question: Past experiences with sharing data with others |
| *1* | N/A | N/A | In general, the cohorts are interested in sharing their data and participating in the analysis. That is more complex with the local processes in some centers: getting the approvals and so on. (…) Main obstacles that we are facing is something which is very important: trust. That those who are sharing the data are always sharing it for collaborative research, that they can trust that the data is treated as it should be treated and I have seen over the years many situations where consortia have disappeared because it appears that they have been doing as well some other analyses on the data without the centers knowing. So trust is key issue and in our case, when we send data to a new place or in particular to some collaborator who is not a member of our consortium, that they hesitate to allow the use, transferring all data, their data to those places. |
| *2* | Yes | Yes | You know data sharing is the main role nowadays and basically the main bulk of the PROJECT X data has been collected based on public funding, [therefore] it is assumed that those data will be public at some states. There are several aspects relevant for this. It is important that those are individual level health data and… There are important privacy issues and bureaucracy related to those privacy issues has become stricter and stricter over the years and it is, it is pretty heavy nowadays actually and the European GDPR is getting problematic or its interpretations are getting problematic… Of course, we fully understand the need of privacy but, but on the other hand, there should be a possibility for data sharing but as far as the research part is involved, the basic rules are that when you do for example laboratory determination for several thousands of individuals which becomes expensive and [unintelligible; getting] to include some research areas, the study group which produces those laboratory results has a certain embargo to use those thousands or all for the [unintelligible; limited] amount of time. For six months or until the first main publication is out. But then, the data goes to a biobank, and is available for other research groups. |
| *3* | Yes | Yes | We share data with X Project of course. We also share data with other EU consortia. In general, in the past only the results were shared but not the actual data. Reanalysis was done at [our institute] and then the results were shared. Nowadays, we also have EU consortia where we also share the data where the data hub is in Country B. That requires a lot of data protection issues, and contractual things but it was all manageable. (…) I don’t expect there will be any problem since it is a GDPR country and they were extremely well organized so that was not really a problem. And the same is for Project X in the end, the rules are very clear, it is within the European Union, so it is all no problem. |
| *4* | N/A | N/A | Specific question not asked: Elements captured over different questions |
| *5* | Yes | Yes | It has been superb. Many thanks to the Project X office in City A and the lead officer there has been excellent, helpful and guiding. Of course, with all these laws and restrictions, it’s like navigating in shallow waters but City A has been extremely helpful and my team here as well. We have come to very good results but of course it took some effort, and lots of papers and lots of signatures and lots of contracts et cetera but I do have a very good supporting organization for that in my university. |
| *6* | Yes | No | Data sharing in X is a very formal process. We post a website, you may apply for data. There is a board that makes a decision on applications and then based on standard contracts, we transfer data or materials. (…) It is very open, and a lot of our success basically depends on these collaborations [internationally]. So, we are favorable towards data sharing with some limitations and these limitations are for instance leaving EU-borders with materials or data because of the more uncertain legal background. |
| 7 | Yes | No | When I started working in X, our cohort data were already transferred in, in City Z. So, I was not involved on the big effort on data standardization and stuff like that. I had only a small role on standardization of follow up data but that is a much easier than baseline standardization so I cannot really tell you my direct experience on, on data sharing. What I have done is lots of analysis of consortia data. That involved from me, trying to understand different cohorts, the data, how they were collected and what are the characteristics of each cohort and that takes a lot of time and… (…) We are also included into genetic consortia and they are much larger. We need to find a right balance because on one side, I feel like I cannot really participate, or fully understand what the analysis are. When I received an already advanced draft paper, I feel like I cannot really have an impact as a co-author on this paper. (…) I receive an advance draft on a very complicated genetic issue with forty tables only in the supplementary material and maybe fifteen days to provide the feedback. This means: Please don’t say anything, just check the affiliation. A different approach would be if I receive in advance a sort of: Hey look we do, we plan to do this, this kind of analysis, in this way you can be more transparent and follow what has been done a little bit better. (…) The only people asking for access so far are the people in the consortium where we are included. We had in the past a couple of, we tried to establish at least a couple of collaborations outside these larger consortia but for one reason or the other, in the end we ended up with pretty much nothing. |
| *8* | Yes | No | Traditionally, we would share anonymized data. (…) What has happened over time because though all the participants have participated to take part in the study, they never consented for us to give their personal details to NHS Digital in terms of data protection act or GDPR. So, we had to get then special permission to continue to have their data. Despite the fact that I can give you anonymous data, it is theoretically possible to re-identify someone if you have enough information about them. Particularly if someone dies from a very rare cause of death, like motor neuron disease and you know the date of death as the first of January, 2005 and you know that this was a man aged 64, it is possible for you to say: Ah, this must be John Smith so therefore you have now reidentified, you may be wrong but you may have reidentified. |
| *9* | Yes | No | We have a lot of genetics consortia involvement at the minute. There’s a lot of genetics projects on the way. And, also the I-health, we do very detailed I-health measurements, so the I-health is another big data area where we’re doing a lot of collaborative work and consortia work in that side of thing. (…) Our biggest problem is overcoming statistical disclosure issues and it’s making sure that the data that we send out isn’t going to identify anybody in any way. That’s our biggest concern and our biggest restriction so it’s all in the GDPR and the Data Protection Regulation that limit us in a way of data sharing. Because once the data is out there, anybody can access it and do you know, do their statistics on it. So you know, we need to be sure, that’s why we started with the safe setting approach so that it’s a controlled environment and so that we are still under control of that data but once we release it, you know it’s maintaining that control over it. Also, a problem with data sharing, is study overlap, it’s keeping track of who’s doing what with the data. So, you could have somebody in America could be doing the same kind of research as somebody here using the X data. So, it’s keeping that collaborative approach but also, allow researchers to do their work independently but it’s that problem of research overlap. |
| *10* | Yes | Yes | It is done continuously so we really participate in many consortia. For example, we have had some projects related to BBMRI, but I don’t even know how many ongoing collaborations are leading currently: too many and we don’t have any restrictions, I think. Any subjective restrictions are that we need to have ethics approval, first for data access and sharing if we need to provide access to some samples. |
| *11* | Yes | Unknown | We have been very keen to share our data with consortia like the X project or also these [unintelligible; WHO?] collaborations going on, a lot of genetic consortia also because we think that when we have used so much effort in collecting these data and when people have been willing to come to us and give us all their information, then it’s also a part of our responsibility to ensure that these data are used. But it is not as easy as it used to be now due to these GDPR rules. Ten years ago, we did need it to have a sign somewhere to be able to send our data, especially if it was to a different country. But I can’t remember that we ever got a no before but right now we don’t even dare asking sometimes because we know it will give us so much work and it has been quite complicated now. |
| *12* | No | No | Specific question not asked: Elements captured over different questions |
| *13* | Yes | Unknown | Let’s say it’s dependent by the consortia of course but major consortium with which we shared our data was really a very good experience was the Z Project that I think generated the project we are speaking about and we had really a very smooth way of collaboration. And we continue to collaborate, also after the end of the project that was financed by the European Community I think 6 or 7 years ago so I don’t remember just when we started. And I think the main reason why this was a smooth process was that all the process was transparent, very clear and all the participants were put on the same level. And this made things quite easy, we had very clear rules for data sharing, for publications, for name on the papers and we feel always good with this process and still now we are fine with this. Of course, this experience was not always the same, we had experiences of other consortium where it was not so simple. The main problem was the way the partner were led to participate in the consortium so in other consortium we had the impression that partners were just providers of data with no possibility to participate actively in the discussion, in originating questions, in analyzing data but all was managed by the coordinators or few people close to the coordinators. So those are the two experiences I have, one very positive and the other one, I cannot say negative but a little bit more difficult. |
| *14* | N/A | N/A | There’s of course all kinds of obstacles along the way. (…) Issues there are of course with the… Do you have to pay for access, that’s an issue where people think the data are there, why should we pay for the data, we try to explain that it costs quite something to set up the structure and to collect data. (…) The issue with sharing is more, let’s say, when we are to combine data with data collected by individual researchers as part of the larger project or their own project. And then there is the idea that they own the data, and that they actually, the particularities of such an individual researcher, that he or she has special requirements before giving access to his or her data, and that depends, it can just being friends or pay for it, or collaborate something or co-authorship. But I guess that you have heard that before. |
| *15* | Yes | Yes | We have participated in quite a large number of European research grants in the Framework Programs and in most of them we have not shared, at least not shared individual data but that has happened also in specific cases where we, where some person in one of the other countries does an analysis and need individual data but usually we have said that, we do not really have the consent from the participants in A to store the individual level data outside the main database in Z. So what we have done is to share aggregated data, for instance genetic data where you do a GWAS and then you sent the aggregated files to for instance the A consortium and the B consortium, which have some kind of a platform, some central facility where data is being collected. But we have said that it’s not possible to put individual level data on any platform. |
| *16* | Yes | No | Since it’s a very general cohort with many health topics addressed, we have many collaborations for the cohorts so a big part of my job is to share data so we share it with teams [of this country] but also within international consortia and for example currently we are involved in the X project and also a more recent one which the Y project, which is H2020 project. |
| *17* | Yes | Unknown | This is already an international project. So, it’s already the conception of the study in itself. I have to backtrack a little bit. This project is a European cohort so it involves 11 countries. So, from the very beginning it was designed to involve data sharing so all the consent participants explain that it was a European project and the whole idea of the project was to see how people do things differently between the different countries of Europe. Since then, I also participate. I also, starting from that project, we put together a platform, much like the platforms you have here, it’s called Z and this brings together similar cohort studies of preterm babies that have been developed in Europe over time and are being put together over time and are being put together on a platform so our European project is participating in this further European project which is federating other cohorts. In our research center, there is also another large national cohort [of this country] which is participating in X. So, I am also involved in facilitating that project although I am not a lead investigator, but I am involved in facilitating making that data available to the international community. And within the context of that project, we have put together several different projects then which involve sharing of data on several different levels, either aggregate data or individual data. |

Interviews are numbered and the sharing of cohorts through international collaborations and biobank is indicated with (YES/NO). Biobank participation was interpreted here as the cohort being part of a formal governance structure that regulates access (e.g. institutional biobanks or cohort-specific system overseen by a board). The quotes are extracted from the answers given by different participants to the question: “What have been your current experiences with sharing data with others? What mode of sharing did you employ?”. In some instances, brackets are used to shorten answers by removing irrelevant sections or to include subsections of follow-up questions.
