# Supplementary material for: Factors that influence data sharing through data sharing platforms: A qualitative study on the views and experiences of cohort holders and platform developers
Source: PLoS One. 2021 Jul 2;16(7):e0254202. doi: 10.1371/journal.pone.0254202 (PMC8253381; doi:10.1371/journal.pone.0254202)
Supplement: S2 File — (DOCX) [file pone.0254202.s003.docx]

**Qualitative study on incentives for data sharing – the views of cohort/data managers and platform developers (VERSION 2)**

**Research question: What are the experiences, views and opinions of cohort/data managers and data sharing platform developers on incentives for data sharing in biomedical sciences?**

**Interview questions:**

1. **General**

- What is your present function? What are your main responsibilities?
- What cohort or platform are you administering?
- Which national/international entities fund your research?

1. **Experiences with data sharing**

- What have been your current experiences with sharing data with others? What mode of sharing did you employ?
- Are data shared only through international consortia or also in a biobank?

1. **Opinion/experiences on data sharing incentives mechanisms**

- What do you consider as the main disincentives/barriers for data sharing in biomedical sciences?
  - Have you experienced problems with the implementation of GDPR?
- How do you think credit for data sharing should be given within data sharing platforms/consortia?
- Do you require co-authorship and an embargo for the sharing of cohort level data of your cohort?
- What is your opinion on extensive authorship lists coming out of international consortia?
  - Do you consider that they complicate the evaluation of productivity of research groups/individuals?
  - How do you decide on authorship where new persons entering the research team have not contributed to the initial collection of the data?
- Do you consider that alternative crediting mechanisms to traditional authorship are viable? Such crediting mechanisms can include contributorship, Data Authorship or the CRediT taxonomy?
- Do you utilize international guidelines for deciding upon who becomes an author?
  - Do you ever deviate from these guidelines?
  - Do you accept the guidelines as they are or would you like to change them?

1. **Opinions and experiences on the role of the various involved parties (funders, journals, institutions etc…)**

- What are the roles of other academic actors, such as funders, academic institutions and journals to ensure that data sharing happens? What role do academic societies have?

1. **Opinions on data sharing and concepts of ownership, control and fairness in data sharing**

- Do you feel a sense of ownership over the data you have generated? What is your view on the argument that the return of investment to society should be maximized?
- Have you personally encountered situations where sharing data has worked to the detriment of your academic/personal career? Could you imagine some instances where this would be the case?
- Did you have cases where there was a misinterpretation of data after not overseeing the research process?
- Do you think the current mechanisms (such as control by Data Access Committees) are adequate in addressing data access control issues?
  - Have you had cases where data requests were made for competing analyses? How did you proceed?

1. **Future of data sharing**

- How do you envision the future of data sharing within the existing academic incentive structure?
- Do you think that certain evolutions on the policy level will change data sharing in the future?
- Do you envision interactions between new technologies and incentives for data sharing?

**Concluding questions**

- Would you like to add any other element that not covered in this interview?
- Thanks!
